# Bottlenecks and Solutions During Implementation of the DREAMS Program for Adolescent Girls and Young Women in Namibia

**DOI:** 10.9745/GHSP-D-22-00226

**Published:** 2022-10-31

**Authors:** Ellen W. MacLachlan, Abigail K. Korn, Alison L. Ensminger, Sharon Zambwe, Theopolina Kueyo, Rosanne Kahuure, Gena Barnabee, Josua Nghipangelwa, Juliet Mudabeti, Prisca Tambo, Agnes Mwilima, Elizabeth Muremi, Norbert Forster, Christa Fischer-Walker, Gabrielle O’Malley

**Affiliations:** a International Training and Education Center for Health, University of Washington, Seattle, WA, USA.; b International Training and Education Center for Health Namibia, Windhoek, Namibia.; c Project HOPE, Katima Mulilo, Namibia.; d Joint United Nations Programme on HIV/AIDS, Windhoek, Namibia.; e Project HOPE, Windhoek, Namibia.; f Ministry of Health and Social Services, Omuthiya, Namibia.; g University of Namibia Neudamm Campus, Windhoek, Namibia.; h Brent Local Authority, London, England.; i Ministry of Health and Social Services, Katima Mulilo, Namibia.; j Formerly of the Ministry of Health and Social Services, Windhoek, Namibia.; k Independent consultant, Edgewood, KY, USA.

## Abstract

**Background:**

We synthesize implementation bottlenecks experienced while implementing the DREAMS (Determined, Resilient, Empowered, AIDS-free, Mentored, and Safe) program, an HIV prevention intervention for adolescent girls and young women (AGYW), in Namibia from 2017 to 2019. Bottlenecks were organized into the following 4 AGYW program components.

**Program Access:**

Enrollment was slowed by the time-intensive nature of screening and other baseline data collection requirements, delays in acquiring parental consent, and limited time for after-school activities. Solutions included obtaining advance consent and providing 1-stop service delivery and transportation assistance.

**Health Education:**

We experienced difficulty identifying safe spaces for AGYW to meet. A lack of tailored curricula also impeded activities. Governments, stakeholders, and partners can plan ahead to help DREAMS identify appropriate safe spaces. Curricula should be identified and adapted before implementation.

**Health Services:**

Uneven availability of government-provided commodities (e.g., condoms, preexposure prophylaxis [PrEP], family planning products) and lack of AGYW-centered PrEP delivery approaches impacted services. Better forecasting of commodity needs and government commitment to supply chain strengthening will help ensure adequate program stock.

**Social Services:**

The availability of only centralized care following gender-based violence (GBV) and the limited number of government social workers to manage GBV cases constrained service provision. Triaging GBV cases—i.e., referring high-risk cases to government social workers and providing DREAMS-specific social services for other cases—can ensure proper caseload management.

**Conclusion:**

These bottlenecks highlight practical implementation issues and higher-level considerations for AGYW-centered HIV prevention programs. The critical need for multilayered programming for HIV/GBV prevention in AGYW cannot be addressed simply with additional funds but requires multilevel collaboration and forecasting. The urgency to achieve results must be balanced with the need for adequate implementation preparedness.

## Abstract (precis)

We analyze implementation bottlenecks experienced in the DREAMS program in Namibia that can provide valuable insights and suggest ways to anticipate and overcome these challenges when managing HIV and gender-based violence prevention programs for adolescent girls and young women.

## Teaser

**Key Findings:**

- Adolescent girls and young women (AGYW)–centered HIV prevention programs, such as DREAMS, must employ layered interventional approaches that address both AGYW health and sociobehavioral issues and must engage stakeholders across multiple sectors, increasing the potential for implementation bottlenecks that can undermine their efficiency and effectiveness.
- Bottlenecks identified involved challenges with program access and enrollment, outdated and unengaging health education curriculum and lack of safe meeting spaces, stock-outs of HIV commodities, and limitations in supporting gender-based violence (GBV) cases.
- Solutions include increasing the government role in identifying safe spaces for AGYW, expanding sexual and reproductive health services for AGYW within existing institutions, ensuring a continuous supply of sexual and reproductive health and HIV commodities, and decentralizing GBV care to increase access.

**Key Implications:**

- These bottlenecks highlight practical implementation issues and higher-level considerations for AGYW-centered HIV/GBV prevention programs.
- The unique programming needs for HIV/GBV prevention in AGYW require multilevel collaboration and forecasting. Successful program implementation necessitates adequate preparation.

## BACKGROUND

Over the past decade, HIV incidence has declined in sub-Saharan Africa, yet remains disproportionately high among females aged 15–24 years, who account for approximately two-thirds of all new infections.^1^^,^^2^ Primary contributing factors include biological risk of HIV infection for adolescent girls and young women (AGYW) because of the permeability of the vaginal epithelial mucosa, sociobehavioral risks due to structural factors, societal power differentials, and gender norms that decrease AGYW agency and result in negative outcomes such as early marriage, sexual relationships with older men, school incompletion, physical and emotional abuse, and unwanted pregnancy.^3^ Age-disparate partnerships in particular have fueled a cycle of HIV transmission from older men to their younger female partners.^4^^–^^9^

In response to calls from the public health community to accelerate HIV/AIDS prevention for AGYW, the DREAMS (Determined, Resilient, Empowered, AIDS-free, Mentored, and Safe) program is being implemented in 15 countries, including Namibia. As a public-private partnership supported by the U.S. President’s Emergency Plan for AIDS Relief (PEPFAR), DREAMS combines multisectoral, layered, evidence-based interventions for AGYW, their sex partners, parents, and the community.^10^^–^^14^

“Layered” here refers to the provision of multiple interventions or services to each AGYW as well as contextual level interventions not delivered directly to an AGYW but from which she may benefit (e.g., changing social norms).^13^^,^^14^ Layered interventions include HIV and gender-based violence (GBV) education for AGYW and their parents, health services, social services such as GBV case management, interventions to change male gender norms, and community mobilization. This layering of multiple interventions has been shown to be highly effective in addressing AGYW health and social issues.^15^^–^^18^

Because of the number of concurrent interventions and components, the DREAMS program must include the active engagement of stakeholders across multiple sectors, such as education, health, social welfare, and safety and security sectors, and are implemented in myriad settings including community centers, schools, churches, and health facilities. The program’s inherent complexities increase the possibility of bottlenecks that can hamper the efficiency of implementation and ultimately undermine program effectiveness, acceptance, and long-term sustainability.^15^

Few published studies of AGYW-centered programs examine and report on the role of bottlenecks in implementation, though key organizations have published guidelines asking for more reporting on implementation challenges.^19^^,^^20^ In response to this call, we describe 2 years of implementation challenges with the DREAMS program in Namibia, including descriptions of bottlenecks faced and how they were addressed.

## IMPLEMENTING DREAMS IN NAMIBIA

**FIGURE 1:**
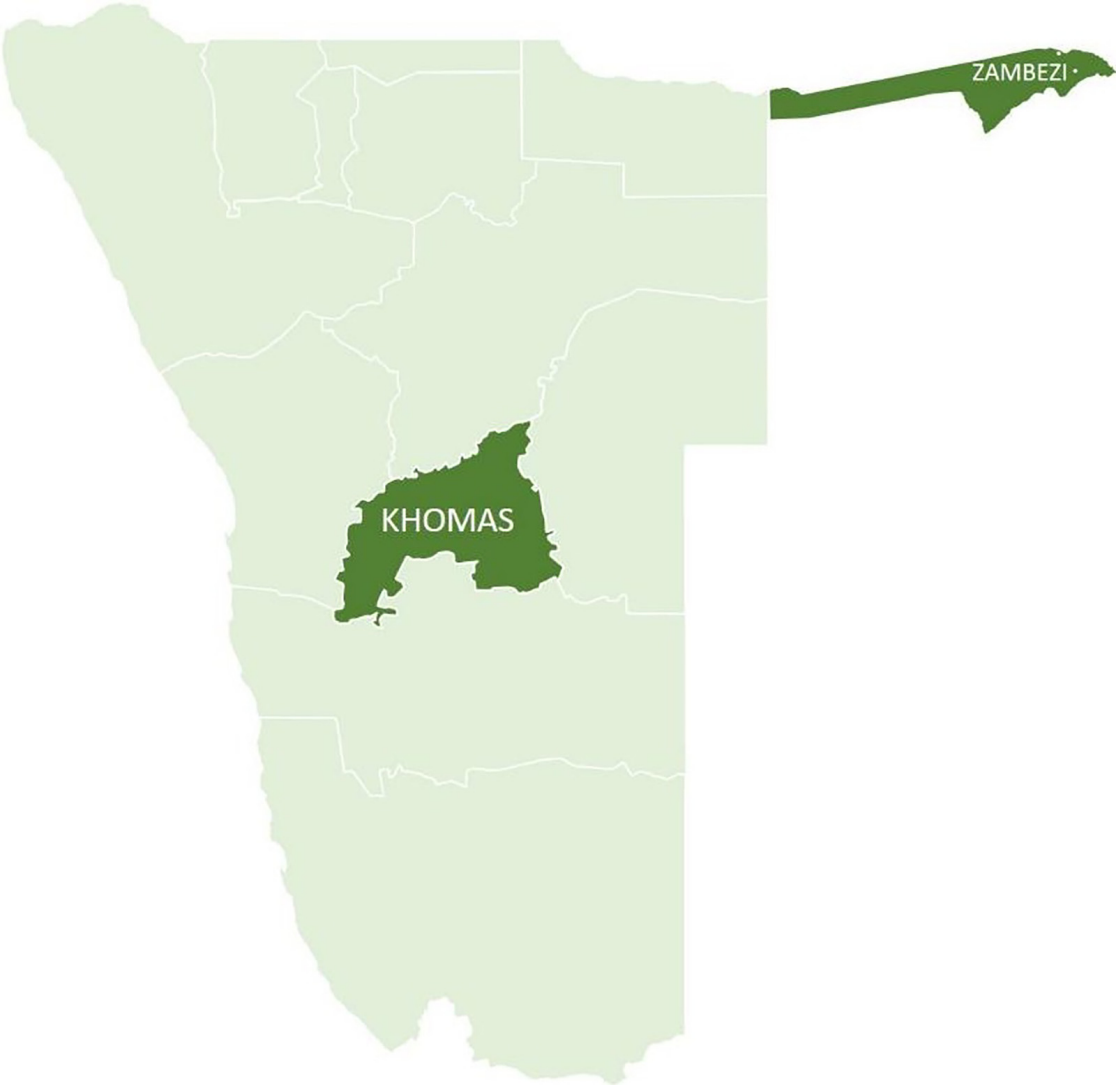
I-TECH DREAMS Regions in Namibia Abbreviations: DREAMS, Determined, Resilient, Empowered, AIDS-free, Mentored, and Safe; I-TECH, International Training and Education Center for Health.

The Namibia DREAMS program launched in 2017 in 2 high HIV prevalence regions, Khomas and Zambezi (Figure 1).^21^^–^^24^ The program was implemented through a multipartner collaboration including the Ministry of Health and Social Services (MOHSS); Ministry of Education, Arts and Culture; Ministry of Gender Equality, Poverty Eradication and Social Welfare; the International Training and Education Center for (I-TECH) at the University of Washington; and partner nongovernmental organizations Lifeline/Childline of Namibia and Star for Life. The program targeted AGYW aged 9–24 years. The information reported here is from the program years 2017 to 2019.

### Program Model

The DREAMS program in Namibia used a safe space model for program and service delivery.^25^ A safe space is a supportive, judgment-free, safe and private location, such as a school classroom, church meeting room, or community center, where AGYW can meet regularly as a group led by a trained peer (or near-peer) mentor.^26^ Over 100 safe spaces were established throughout both regions, each with a peer mentor assigned to meet once or twice a week with 20–30 AGYW grouped by age: 9- to 14-year-olds, 15- to 19-year-olds, and 20- to 24-year-olds. All AGYW and program staff, including the peer mentor, traveled to the safe space. School-based safe spaces did not require as much travel for AGYW compared to other safe spaces (e.g., community centers, churches, and health centers). Since AGYW were in the safe spaces for 2–3 hours or more, a small meal and drink were provided.

DREAMS in Namibia used a safe space model for program and service delivery where AGYW meet in a supportive, judgment-free, safe, and private location.

### Recruitment

**FIGURE 2:**
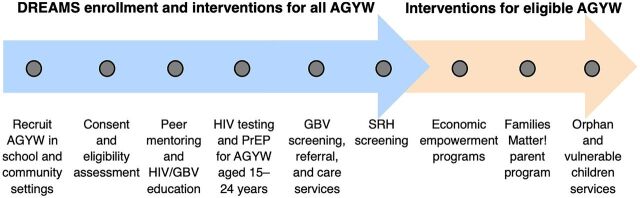
Flowchart of DREAMS Enrollment Activities and Interventions for All AGYW, and Additional Interventions for Eligible AGYW Based on HIV/GBV Risk in Namibia Abbreviations: AGYW, adolescent girls and young women; DREAMS, Determined, Resilient, Empowered, AIDS-free, Mentored, and Safe; GBV, gender-based violence; PrEP, preexposure prophylaxis; SRH, sexual and reproductive health.

Recruitment was organized by age group. Participants aged 9–14 years were recruited almost entirely from primary schools. Girls aged 15–19 years were recruited from secondary schools, house-to-house engagement, special events, and health facilities. Young women aged 20–24 were recruited from tertiary education settings, house-to-house engagement, special events, and health facilities. In addition to providing classroom space, teachers and school administrators were involved in helping communicate with parents, assisting AGYW who experienced violence, and providing insight into the HIV/GBV education curricula, as many teachers had taught the same content to students. Recruitment was driven by PEPFAR targets for DREAMS (approximately 16,000 AGYW enrolled each year), which aimed to reach “saturation” whereby 75% of vulnerable AGYW aged 9–24 years in a district or region complete an age-specific primary package of DREAMS interventions and need-based secondary interventions (Figure 2).^27^

### Enrollment

Enrollment procedures included consent, collection of basic demographic information, and an HIV/GBV risk and vulnerability assessment that was introduced 1 year into program implementation. All AGYW provided written consent before participation. Written parental consent was obtained from parents of AGYW aged 9–19 years regardless of setting (young women aged 18–19 in schools were asked for parental consent because of the Ministry of Education, Arts and Culture policies and the program decided to require the same for out-of-school AGYW to maintain program consistency). To reach the most vulnerable AGYW, and as required by PEPFAR, program staff administered an HIV/GBV risk and vulnerability assessment to each AGYW, with responses determining eligibility for secondary DREAMS interventions.

### Services

Regardless of risk assessment results, all AGYW received basic HIV/GBV prevention education and health and social services (blue arrow in Figure 2) while eligible AGYW received secondary interventions based on need (tan arrow in Figure 2). When meeting at the safe spaces, AGYW received regular HIV/GBV prevention education sessions led by peer mentors and, on select days, GBV services (psychosocial interventions and postviolence care) and sexual and reproductive health (SRH) and HIV prevention services (i.e., HIV testing, preexposure prophylaxis [PrEP], family planning). These services were provided by DREAMS program social workers, lay counselors, and health care providers (certified HIV testers and registered nurses). The primary package of services varied by age group. Program teams could visit 2–3 safe spaces in a day, providing services to nearly 100 AGYW in a single day.

All AGYW received basic HIV/GBV prevention education and health and social services while eligible AGYW received secondary interventions based on need.

### Staffing

To implement DREAMS, the program employed 105 full-time staff including program managers, nurses, HIV testers, lay counselors, social workers, drivers, monitoring and evaluation (M&E) staff, and over 200 part-time staff (i.e., peer mentors, peer mentor supervisors, and community recruiters). The total implementation cost of DREAMS in the 2 regions over 2 years was approximately $US3.2 million. I-TECH established a separate DREAMS office in each region where staff were organized by team: M&E, social services, and health services. Each regional office oversaw a large team of part-time staff who were entirely community-based. Each region had 2 vehicles.

### Data Collection and M&E

A monitoring system was designed to capture data required for PEPFAR reporting and to support program quality and continuity of services. Data were collected at enrollment and at each point of service delivery and managed using Research Electronic Data Capture (REDCap).^28^^,^^29^ Attendance by AGYW at health education sessions was collected on paper by peer mentors and entered in REDCap by M&E staff. M&E staff also administered both a baseline questionnaire and the HIV/GBV risk assessment questionnaire to all AGYW at enrollment. Data on layering were collected (i.e., the number of services each AGYW received) but are not reported here because the service definitions varied considerably during this 2-year start-up phase of DREAMS in Namibia.

### RESULTS

Within 18 months, the DREAMS program under I-TECH enrolled 20,150 participants, approximately 26% of the AGYW population living in the Khomas and Zambezi regions according to the 2011 national census.^30^ The program was able to provide HIV prevention services to 84% of participating AGYW (16,926) and 75% of AGYW (15,113) received both health and social services; other AGYW did not receive these services but did receive at least 1 service from DREAMS. All AGYW who reported high-risk GBV and the majority of low- and medium-risk GBV cases received postviolence care services. Once an AGYW was enrolled in DREAMS, retention was measured by attendance at safe space sessions with her peer mentor. Attendance at half or more of HIV/GBV prevention education sessions was higher in the Zambezi region (76.5% attendance), a more rural region with a limited offer of alternative activities for AGYW, compared to Khomas region (52.9% attendance) where the capital Windhoek is located. In both regions, girls aged 9–14 years recorded the highest attendance (68.2% overall) possibly due to the great enjoyment of DREAMS observed in this age group and their limited access to alternative activities.

## BOTTLENECKS AND SOLUTIONS

We report bottlenecks experienced during the implementation of 4 different program components for DREAMS in Namibia: program access, health education, health services, and social services (Table 1) and then highlight solutions found to overcome these bottlenecks (Table 2). These bottlenecks and solutions are derived from program staff meetings during the time period and observations during field visits by all program staff.

**TABLE 1.** Key DREAMS Bottlenecks in Namibia

**Program Component**	**Bottlenecks**
Program access	Limited availability of AGYW for enrollment after school let out Considerable time needed to administer baseline and HIV/GBV risk forms Requirement for written parental consent for almost all AGYW (aged 9–19 years) added several weeks to the preparation time for beginning activities Requirement for constant Internet access and equipment to review records to identify duplicate IDs Considerable time needed to develop formal agreements with colleges to allow for DREAMS recruitment reduced program access for these AGYW
Health education	Difficulties identifying and establishing safe spaces for peer mentoring Labor-intensive tracking of attendance for funder reporting reduced time for improving program quality Outdated curricula and AGYW reports that format was insufficiently engaging Variable levels of prior training and facilitation experience of peer mentors who delivered sessions and mentors were often in their first professional position Delayed consensus on what curriculum to use for women aged 20–24 years
Health services	Stock-outs of commodities during implementation Challenges and considerable time to transition to implementation of AGYW-preferred community-based PrEP follow-up versus follow-up at clinics Lack of Government of Namibia–finalized national PrEP implementation plan (at the time) Refusal of some schools to allow family planning services for AGYW and lack of sexual and reproductive health in school health policy Lack of communication to all levels of government about DREAMS program objectives
Social services	Insufficient number of MoGEPESW staff to handle GBV referrals from DREAMS Considerable access issue for postrape care in Namibia, which is centralized at GBV units located only at large tertiary hospitals Considerable time and effort to transport AGYW to GBV units and to provide other assistance Substantial staff time needed to implement psychosocial interventions and counseling for any AGYW that screened positive for GBV Lack of dedicated space to provide confidential psychology counseling to AGYW

Abbreviations: AGYW, adolescent girls and young women; DREAMS, Determined, Resilient, Empowered, AIDS-free, Mentored, and Safe; GBV, gender-based violence; MoGEPESW, Ministry of Gender Equality, Poverty Eradication and Social Welfare; PrEP, preexposure prophylaxis.

**TABLE 2.** Summary of DREAMS Program Planning and Preparation Needs by Program Component

**Program Component**	**DREAMS Program Planning and Preparation Needs**
Program access	Initiate enrollment in community and at schools before program start; plan with schools for enrollment during school hours and use community special events and/or one-on-one house visits to speed enrollment. Consider online enrollment if feasible. Work with schools to allow self-consent for students aged 18 years or older. Plan ahead to efficiently obtain parental consent. Expect resource and logistics complications relating to Internet, food, child care, transportation, and staffing. Work in advance to allow program access to eligible AGYW in tertiary education settings.
Health education	Plan strategically with ministries, the private sector, NGOs, churches, and local authorities to identify an adequate number of appropriate safe spaces. Identify staff to manage most administrative tasks, such as program tracking, ensuring peer mentors can focus on their primary role and M&E staff can be free up to focus on data quality. Identify evidence-based, locally adapted, and developmentally appropriate HIV/GBV prevention curricula before program start or add content to existing curricula while awaiting approved content.
Health services	Carefully evaluate which PrEP model will work best for AGYW in your particular DREAMS setting and plan ahead for logistics. Consider that many AGYW do not prefer to access PrEP at health facilities. Work with government supply chain offices to anticipate increases in stock use (e.g., HIV commodities and family planning) with onset of DREAMS program. Clearly communicate health services components of DREAMS to all stakeholders, especially HIV testing, PrEP, and family planning. AGYW program donors need to advocate for national school health policies that include provision of sexual and reproductive health services.
Social services	Consider government managing high-risk GBV cases and DREAMS managing medium- and low-risk cases. Anticipate a high GBV case load and consider cadres to help manage cases (e.g., lay counselors and part-time social workers), in addition to full-time social workers. Work with stakeholders early in the planning process to explore possibilities for expanded post-GBV service delivery sites. Provide staff with trauma counseling training. Identify private spaces where AGYW can receive psychological counseling. Participate in advocacy efforts related to HIV/GBV linkages.

Abbreviations: AGYW, adolescent girls and young women; DREAMS, Determined, Resilient, Empowered, AIDS-free, Mentored, and Safe; GBV, gender-based violence; NGO, nongovernmental organization; PrEP, preexposure prophylaxis.

### Program Access Bottlenecks

Although gathering AGYW in safe spaces was logistically easier in school settings, it was challenging to screen and enroll hundreds of AGYW at the end of the school day, especially when AGYW had to walk long distances home before dark for their safety (Table 1). Gathering written parental consent forms in person from parents for AGYW aged 9–19 years was required because of government regulations and often took 2–3 weeks; AGYW whose parents could not be reached could not participate. Enrollment sessions also required extensive data collection, such as demographic information and risk/vulnerability screenings, which required substantial staffing, typically 10–15 staff per session. To ensure AGYW were not enrolled in more than 1 space, Internet connectivity was needed for M&E staff to check new enrollees against existing records using a table or laptop computer for access to this database. Lastly, to enable enrollment of 20- to 24-year-olds attending tertiary education institutions or technical training colleges, program leadership spent many months developing and formalizing partnerships (e.g., memoranda of understanding) allowing participation in DREAMS, which delayed recruitment of this age group (Table 1).

### Program Access Solutions

#### Limited Time for Enrollment After School

Preprogram planning with school and community group representatives identified alternative time periods for enrollment of AGYW, such as during the school day or during special school or community events. Programs could also explore one-by-one DREAMS enrollment during door-to-door recruitment in the community, special events, or automatic enrollment when students enroll in school (Table 2).

#### Labor-Intensive Data Collection

Program staff and time burdens were addressed through the introduction of self-administered risk/vulnerability assessments for older AGYW aged 20–24 years. In some settings, self-administered online enrollment could also be utilized. DREAMS programs should carefully consider the added value of all preenrollment procedures given the time and effort involved in the field and burden on AGYW (Table 2).

DREAMS programs should carefully consider the added value of all preenrollment procedures given the time and effort involved in the field and burden for AGYW.

#### Written Parental Consent

Electronic written consent, through WhatsApp or a similar platform, or utilizing verbal consent procedures given the low-risk nature of the program, could be explored. Participation of AGYW aged 18 years or older (legal adults) should not require parental consent since this is the age of majority, even in school settings. In Namibia, this requirement sometimes prevented inclusion of AGYW most in need of services as older adolescents often live in school dormitories away from home, especially in rural areas (Table 2).

#### Limited Access for the 20- to 24-Year-Old Age Group

This bottleneck was addressed partly through organization of 1-stop DREAMS events where AGYW in this age group could receive all the services available. To further aid with access, the DREAMS program organized transportation for this age group and provided babysitting services in addition to small snacks and drinks. Colleges, universities, and training institutions should plan in advance to allow for DREAMS access for AGYW enrollees aged 20–24 years (Table 2). Also, there was noticeable lack of HIV and SRH services available to students at these institutions.

### Health Education Bottlenecks

Although there were Government of Namibia–approved curricula available for HIV/GBV prevention for 9- to 14-year-olds and 15- to 19-year-olds, neither curriculum had been updated for at least 5 years (Table 1). Updating these materials with current evidence-based HIV and GBV content required significant modifications. AGYW aged 15–19 years and their peer mentors reported that the didactic format of their curriculum was dull and unengaging, which may have contributed to low attendance. This was especially problematic in the Khomas region where attendance in and around Windhoek was already hampered by distance and safety concerns. No curriculum existed for AGYW aged 20–24 years and the considerable amount of time it took to identify an evidence-based curriculum for this age group that was acceptable to all stakeholders slowed implementation.

With thousands of AGYW at over 100 safe spaces, attendance tracking required for reporting purposes was an especially time- and labor-intensive process. It took up valuable time for all team members, especially peer mentors and M&E teams, and came at the expense of other M&E processes needed for improving program quality (Table 1).

Employment as a peer mentor was often the first formal employment experience for the young women hired into this role. Even though all peer mentors were fully trained to deliver the education sessions, their skill and competence varied widely, requiring considerable ongoing training, support, and supervision to maintain a high teaching standard and ensure ongoing attendance reporting (Table 1).

### Health Education Solutions

#### Identifying Safe Spaces

Many of the challenges in implementing health education related to identifying safe spaces that were private and safe, affordable, and suitable for the required activities. Program staff need to conduct advance strategic planning with ministries, the private sector, nongovernmental organizations, churches, and local authorities to identify an adequate number of appropriate safe spaces for DREAMS meetings (Table 2).

#### M&E Burden on Peer Mentors and M&E Team

Removing attendance tracking and administrative tasks from peer mentors, or simplifying them with better technology, would enable peer mentors to focus their time and energy on teaching and mentoring AGYW, improving health education delivery, and allowing for expansion of the peer mentoring component. In addition, a frank discussion about the value of the extensive DREAMS M&E is also needed. Simplifying attendance reporting also frees up time for the M&E team to focus on data quality and analysis of data for improving the program (Table 2).

#### Appropriate Curricula

DREAMS programs need considerable advanced planning with appropriate ministries and other stakeholders to be able to identify and/or adapt an acceptable curriculum for each age group (Table 2). Success depends on having an evidence-based, localized curriculum that can keep the interest and engagement of AGYW and can be readily adapted—an overly didactic curriculum will negatively impact attendance. The DREAMS team added activities to existing curricula that were fun, engaging, and technically up-to-date while waiting for new evidence-based and stakeholder-approved curricula. Finally, in Namibia we found there were fewer options for previously vetted curricula for the 20- to 24-year-old age group compared to younger age groups; it is particularly important to identify appropriate HIV/GBV prevention curricula for this older age group early in program planning.

### Health Services Bottlenecks

Although PrEP was included in the Namibia 2016 National Guidelines for Antiretroviral Therapy, development of the Government’s PrEP implementation plan launched concurrently with the DREAMS program, leaving DREAMS without an existing national PrEP delivery plan to follow.^31^ As a result, many aspects of PrEP delivery, including training of health care workers and health communication activities such as flyer and poster development, became the responsibility of DREAMS (Table 1). Before DREAMS, PrEP services were delivered entirely within health facilities; however, AGYW reported that they preferred not to obtain PrEP at government facilities due to some health workers’ lack of discretion and unwelcoming attitudes.

DREAMS relied on the Namibian government’s commodities such as condoms, HIV test kits, family planning methods, and PrEP, which are distributed to public health facilities by the MOHSS Central Medical Stores. For various reasons, including a severe economic contraction in the country, these commodities were at times unavailable for long periods. Stock-outs not only delayed DREAMS health services but also complicated follow-up services for AGYW, especially family planning and PrEP. Other complications included the refusal of some schools early in the implementation of DREAMS to allow family planning services to be delivered in the school setting. While family planning service delivery in schools was under discussion between stakeholders, the program provided these services to AGYW in community settings only.

### Health Services Solutions

#### PrEP Service Delivery

The DREAMS program introduced innovative AGYW-friendly PrEP services in Namibia. In Khomas, PrEP service delivery successfully transitioned from an initial hybrid community-clinic model where AGYW were referred to government facilities for refills to a wholly community-based model, even though the transition was time-intensive and logistically challenging. In Zambezi region, where PrEP services began after those in Khomas, a community-based PrEP model was successfully implemented from the onset by applying lessons learned in Khomas. DREAMS and other similar programs for AGYW should consider different models for delivering PrEP to AGYW and organize strong support for selected models before launching the service (Table 2).

In both regions, PrEP service delivery for AGYW successfully transitioned from a community-clinic model to a wholly community-based model.

#### Stock-Outs

Delivering services to thousands of AGYW may well put an additional strain on an already undersupplied health sector. Ongoing coordination and collaboration are needed to anticipate commodity supply requirements and ensure that government forecasting can fully account for the increased demand from DREAMS (Table 2). It may also be possible to plan for a DREAMS-specific stock to be set aside from the beginning of the program given that, at times, realistic stock demand is difficult to quantify. Ongoing government commitment, resources, and health systems strengthening are needed to ensure adequate DREAMS stock, which will greatly contribute to the program’s sustainability.

#### School-Based Delivery of Services

All DREAMS stakeholders, especially ministries of education, need to understand and communicate support for DREAMS services from national to regional and local levels to avoid possible misunderstandings. Since national school health policy at the time did not explicitly mention SRH services as part of the range of health services to be offered at schools by the MOHSS, PEPFAR and other AGYW program donors need to advocate for national school health policies that include provision of SRH services so that interventions designed to meet the SRH needs of AGYW in school settings can be legally implemented (Table 2).

### Social Services Bottlenecks

The Ministry of Gender Equality, Poverty Eradication and Social Welfare, which is responsible for managing GBV cases in Namibia, was often too understaffed to manage the high number of cases identified through DREAMS (Table 1). While the social workers on staff were able to provide services to AGYW most in need, the caseload far surpassed existing staff capacity. In addition, Namibian health policy prohibits health facility clinicians from attending to rape cases unless adequately trained, therefore necessitating the management of these cases by GBV units at the referral hospital level. When DREAMS staff identified a rape case, they offered to transport the survivor to the hospital and help her register at the GBV unit. Support for rape cases included providing initial first-line trauma counseling and at times calling the GBV unit to confirm availability of the social worker, contacting the AGYW’s family (or friend), or contacting the police. As a result of its time-sensitive nature, support for rape victims took immediate precedence over other GBV counseling and case management activities for AGYW in DREAMS, often requiring considerable time and effort from the program social workers (Table 1).

Finally, the DREAMS program did not have enough private, confidential spaces to provide in-depth psychological counseling to many AGYW. This lack of privacy made it difficult for AGYW to discuss their GBV experiences with program staff. In such cases, the sessions were delayed until a private space could be identified (Table 1).

### Social Services Solutions

#### High GBV Caseload

Over time, the program addressed the issue of the high GBV caseload by having the Government manage only the most time-sensitive GBV cases, such as imminent rape cases. Other GBV cases, including prior rape cases that had already received services by the relevant ministry but where the AGYW needed more care and follow-up, were managed by DREAMS social workers and lay counselors. This triaging of cases helped the program manage the GBV caseload (Table 2).

#### Centralized GBV Care

Multisectoral involvement and coordination are essential to create awareness of and appropriately plan for the expected increase in demand for GBV services under DREAMS. Involving stakeholders early in the planning process for GBV programming could circumvent unnecessary obstacles such as limiting postrape services to difficult-to-reach locations (e.g., at distant tertiary hospitals). It is critical that post-GBV care is decentralized from tertiary care centers to more peripheral levels in Namibia to help increase access to postviolence (Table 2).

Multisectoral involvement and coordination is essential to create awareness of and appropriately plan for the expected increase in demand for GBV services under DREAMS.

#### Limited Training to Support GBV Cases

Social workers and other staff should receive training on trauma counseling in anticipation of managing cases of rape, incest, drug addiction, human trafficking, and severe emotional and physical abuse, within the context of the LIVES approach (Table 2).^32^ Indeed, PEPFAR and the U.S. Centers for Disease Control and Prevention funded health care provider training in this approach in 2019–2020 to expand the staff able to care for GBV cases.

#### Requirements for High-Quality Social Services Delivery

To truly take on the individual and group GBV services needed, DREAMS or any AGYW program manager needs to anticipate the considerable staffing, training, and infrastructure needed (Table 2). This includes the availability of private GBV counseling spaces, similar to what is required for HIV counseling and testing. Well-thought-out advocacy efforts by DREAMS programs can continue to increase awareness of the interconnectedness and extent of HIV/GBV linkages.

## CONCLUSIONS

Our program results show the substantial reach of the DREAMS program in Namibia and the significant demand for AGYW-specific services. AGYW loved the opportunity to participate in DREAMS because there are often few other fun activities available for preteen girls, teen girls, and young women where they can interact openly and freely with their peers and near-peer mentors. Such demand has been documented in other high HIV prevalence countries.^33^^–^^36^ At the same time, peer mentors and other staff observed that some content was unengaging (e.g., HIV/GBV prevention education curriculum)—a somewhat contradictory result—and so staff bolstered curricular content with additional activities and lessons.

Previously published articles have reported on DREAMS topics such as HIV/GBV prevalence, characterization of male partners of AGYW, and how to rigorously evaluate DREAMS and AGYW PrEP persistence, but few have reported on the implementation challenges associated with launching and establishing a complex new multisectoral combination prevention program.^37^^–^^42^ Chimbindi et al. reported on challenges in implementing DREAMS in Kenya, South Africa, and Zimbabwe that mirrored those we have reported here: the urgent and ambitious expectations of DREAMS; layering multiple interventions across different sectors (health, education, and social welfare); and the need for supporting individuals’ transport between services to improve uptake and retention.^42^ In Namibia, we similarly found that expectations of the DREAMS program (and PEPFAR) were “ambitious and bold to implement and achieve impact in a quick time frame.”^42^ Also, this ambitious time frame left little time for troubleshooting implementation bottlenecks during the first 2 years of programming. The planning, organizing, coordinating, and monitoring required before DREAMS program launch, and immediately afterward, were in constant tension with the expectation to achieve PEPFAR targets by the first semiannual period.

Anticipating many of the bottlenecks and potential solutions we describe (also summarized in Table 2) can aid in increasing the sustainability of a program such as DREAMS. For example, increasing the government role in identifying safe spaces for AGYW, expanding SRH services for AGYW within existing institutions (e.g., high schools, tertiary educational institutions, and technical schools), ensuring a continuous supply of SRH and HIV commodities, and decentralizing GBV care are all interventions that could expand critical services to AGYW without having to rely on implementation of a costly program such as DREAMS.^43^ The bottlenecks and solutions described here are not unique to DREAMS and likely apply to other combination prevention programs for AGYW.

The bottlenecks and solutions described here are not unique to DREAMS and likely apply to other combination prevention programs for AGYW.

We do not report here on the number of services AGYW received, as the definition of each service changed considerably over the 2-year implementation period, but this measurement is critical for AGYW programs since they are more likely to benefit if they receive 3 or more services.^26^

Effective HIV/GBV prevention programs for AGYW are multicomponent, layered programs that respond to the multidimensionality of localized influences on AGYW health-related behaviors. Given the complex nature of AGYW programs, a variety of implementation bottlenecks are inevitable. These challenges can be overcome with iterative innovation and flexibility and by planning well in advance for contingencies related to probable future difficulties. Programs for AGYW should continue to document implementation challenges and bottlenecks so lessons learned can be widely shared.

## Notes

**Peer Reviewed**

**First published online:** October 14, 2022.
